# Single-cell RNA sequencing in diffuse large B-cell lymphoma: tumor heterogeneity, microenvironment, resistance, and prognostic markers

**DOI:** 10.3389/fonc.2025.1583250

**Published:** 2025-04-09

**Authors:** Linwei Li, Qiwei Li, Rui Niu, Wei Sun, Hua Liang

**Affiliations:** ^1^ Heilongjiang University of Chinese Medicine, Harbin, China; ^2^ Heilongjiang Provincial Hospital, Harbin, Heilongjiang, China; ^3^ Central Hospital of Siping City, Siping, China

**Keywords:** single-cell RNA sequencing, diffuse large B-cell lymphoma (DLBCL), tumor heterogeneity, microenvironment, prognostic biomarkers

## Abstract

Diffuse large B-cell lymphoma (DLBCL) is a highly heterogeneous malignancy with challenges in treatment resistance and relapse. Single-cell RNA sequencing (scRNA-seq) has provided important insights into tumor heterogeneity, microenvironment interactions, resistance mechanisms, and prognostic biomarkers. This review summarizes key findings from scRNA-seq studies, which have deepened our understanding of DLBCL and contributed to the development of precision therapeutic strategies. Integrating scRNA-seq with spatial transcriptomics and single-cell multi-omics may further elucidate disease mechanisms and identify novel therapeutic targets, supporting the advancement of precision medicine in DLBCL.

## 1 Introduction

Diffuse large B-cell lymphoma (DLBCL), the most prevalent hematologic malignancy in adults, constitutes approximately 35% of non-Hodgkin lymphoma cases (1, 2). Epidemiologic data from the U.S. Cancer Registry reveal an age-standardized incidence rate of 7.2 per 100,000 individuals, with a pronounced male predominance and a clear correlation between advancing age and disease occurrence (3). A significant rise in cases has been noted in areas with historically low incidence rates of DLBCL, despite the fact that the general incidence has remained largely unchanged.

The cornerstone of first-line treatment continues to be the R-CHOP regimen, which consists of vincristine, doxorubicin, cyclophosphamide, prednisone, and rituximab. Although it has significantly improved survival outcomes, approximately 50% of patients experience refractory or relapsed disease following treatment (4–6). The prognosis for patients with refractory DLBCL is poor, with overall survival rates around 20% (7, 8). Chimeric antigen receptor T-cell (CAR-T) therapy has become a viable therapeutic strategy in recent years (9). However, the persistent problems of resistance and recurrence underscore the urgent need for a deeper comprehension of immune escape mechanisms and tumor biology (10, 11).

## 2 Single-cell RNA sequencing: reshaping cancer biology

Single-cell RNA sequencing (ScRNA-seq) is a transformative high-throughput technology that enables high-resolution analysis of gene expression, epigenetic modifications, and intercellular interactions at the single-cell level, offering significant advantages over traditional bulk sequencing (12–16).

In cancer research, scRNA-seq has been widely used to characterize tumor heterogeneity (17–19), identify key gene expression signatures (20–22), and elucidate their roles in tumor progression, metastasis, and drug resistance (17–19, 23). It also enables precise mapping of the tumor immune microenvironment (24, 25), revealing immune cell composition, functional states, and tumor-immune interactions, thereby advancing immunotherapy strategies (26–28). Beyond oncology, scRNA-seq provides critical molecular insights into cardiovascular diseases, muscle development, autoimmune disorders, and tissue regeneration. It has delineated the transcriptional landscape of hypertrophic cardiomyopathy (29), uncovered alternative splicing dynamics in muscle cells (30), and contributed to the study of autoimmune diseases, skin wound healing, and animal physiology (31–34). Moreover, iMLGAM integrates scRNA-seq with machine learning to predict ICB outcomes, offering significant potential for advancing precision medicine (35). Overall, scRNA-seq has significantly contributed to biomedical research, providing a valuable tool for understanding disease mechanisms and supporting precision medicine advancements.

The scRNA-seq enables high-resolution analysis of cellular heterogeneity by isolating individual cells, capturing RNA, and sequencing transcriptomes to generate gene expression profiles (36). Recent advances, such as droplet-based systems (e.g., 10x Genomics) and combinatorial indexing, have enhanced scalability and cost-efficiency, enabling large-scale studies. However, challenges remain, including low capture efficiency, amplification bias, data sparsity, and batch effects, which limit the analysis of low-abundance transcripts. Additionally, scRNA-seq fails to preserve spatial relationships and microenvironmental context, which spatial transcriptomics can partially address (37).

**Figure 1:**
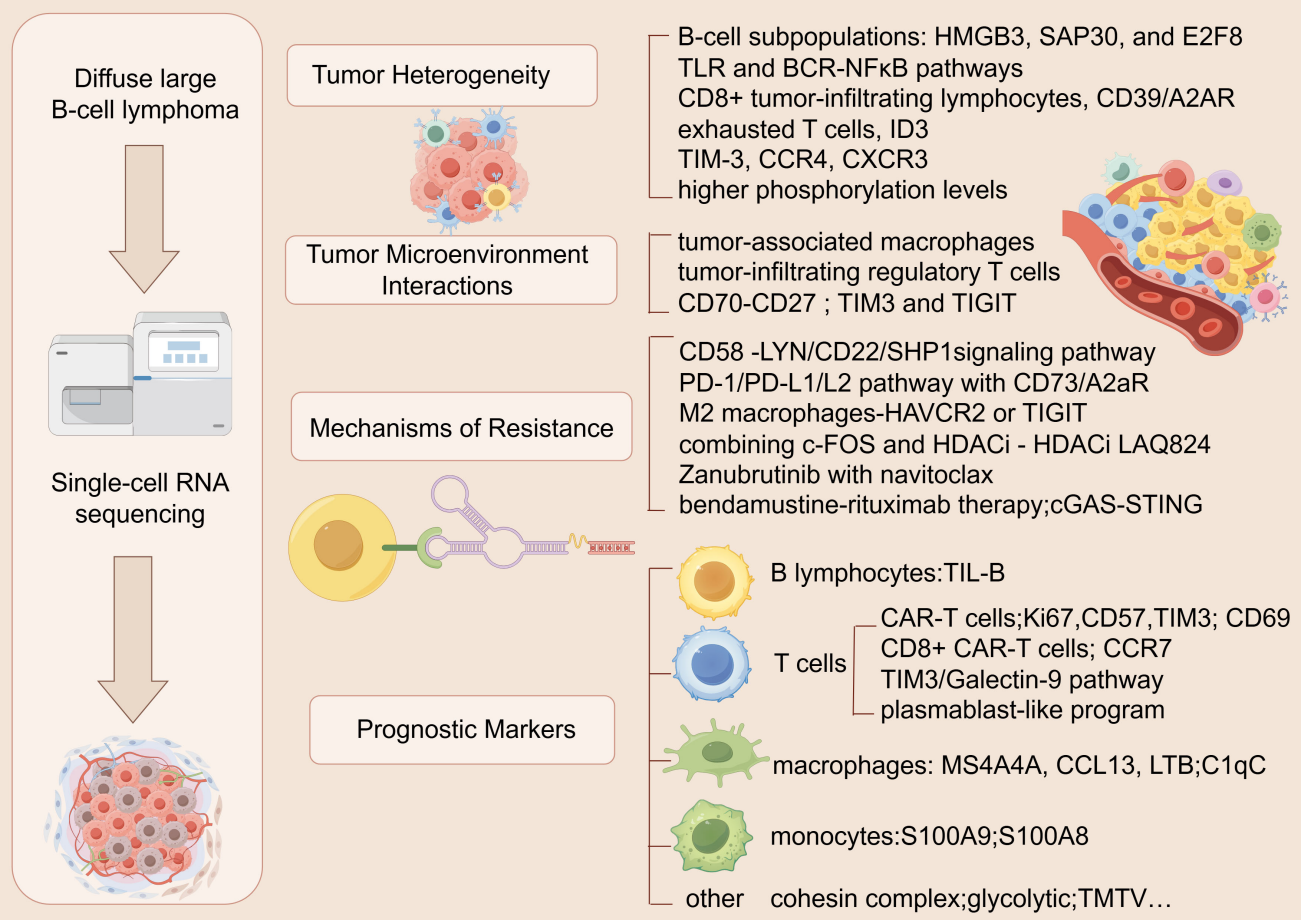
Application of scRNA-seq in DLBCL.

In DLBCL, scRNA-seq has identified key cell subpopulations (e.g., cancer stem cell-like B cells and exhausted T cells), elucidated microenvironment dynamics, and uncovered mechanisms of immune evasion and therapy resistance (38–41) (**Figure 1**). Despite challenges like technical biases and data integration, scRNA-seq holds immense potential for discovering prognostic biomarkers and therapeutic targets. Integrating scRNA-seq with multi-omics and spatial transcriptomics will further advance precision medicine, improving DLBCL diagnosis, treatment, and patient outcomes.

## 3 ScRNA-seq in DLBCL: key insights

### 3.1 Tumor heterogeneity

ScRNA-seq has played a pivotal role in uncovering the tumor heterogeneity of DLBCL. Fengling Liu et al. conducted a single-cell analysis of B-cell subpopulations in DLBCL and normal lymphoid tissues, revealing that the high infiltration of cancer stem cell-like B-cell subpopulations is significantly associated with poor patient prognosis. They discovered a critical transcription factor network controlled by HMGB3, SAP30, and E2F8, and confirmed the existence of this subpopulation through immunofluorescence assays (38). Shu Wang et al. examined the heterogeneity between primary central nervous system DLBCL and extracerebral DLBCL at both the transcriptomic and genomic levels, revealing that mutations in the TLR and BCR-NFκB pathways are critical drivers of malignant B-cell proliferation within the central nervous system (42).

In terms of immune cell functional heterogeneity, Qiqi Zhu focused on the exhaustion heterogeneity of CD8+ tumor-infiltrating lymphocytes (TILs) in DLBCL, revealing that exhausted CD8+ TILs comprise both precursor and terminal states. She also identified that the CD39/A2AR pathway may drive the exhaustion process of these TILs (43). By analyzing single-cell databases in DLBCL, Zhencang Zhou discovered a significantly higher proportion of exhausted T cells in DLBCL compared to normal tissues, along with a notable upregulation of the ID3 gene in these exhausted T cells (44). Furthermore, Anthony R. Colombo et al. compared the expression of immune checkpoint molecules, such as PD-L1, PD-1, and TIM-3, in Hodgkin lymphoma and DLBCL using imaging mass cytometry and single-cell spatial analysis. Potential therapeutic targets like TIM-3, CCR4, and CXCR3 were found via their study (45).

In terms of signal transduction, a study utilized phospho-specific flow cytometry to analyze the B-cell signaling profiles of non-Hodgkin lymphoma patients. The researchers found that lymphoma cells from DLBCL patients exhibited higher phosphorylation levels at multiple signaling nodes, a significant distinction from those observed in mantle cell lymphoma (46). These findings emphasize the potential of targeted therapies that tackle tumor cell heterogeneity. Future advancements in single-cell technologies may further refine treatment strategies and improve prognostic accuracy in DLBCL.

### 3.2 Tumor microenvironment interactions

Tumor heterogeneity in DLBCL shapes a dynamic interplay between tumor cells and immune cells within the microenvironment. Single-cell analysis has revealed that diverse tumor subpopulations influence the microenvironment, contributing to immune evasion, tumor progression, and resistance to treatment. These subpopulations interact with immune cells and stromal components, creating a complex microenvironment that drives disease progression. ScRNA-seq has provided key insights into these intercellular interactions, emphasizing the importance of understanding tumor-immune cell dynamics for developing more effective therapeutic strategies in DLBCL.

Benoit Manfroi et al. found through single-cell sequencing that tumor-associated macrophages (TAMs) in DLBCL exhibit atypical genetic characteristics and express both M1 and M2 macrophage marker genes, which may explain the lack of significant influence of TAMs in DLBCL progression (47). Additionally, studies have shown that highly immunosuppressive activated tumor-infiltrating regulatory T cells (Tregs) are significantly enriched in DLBCL tissues, suggesting that they may play a crucial role in tumorigenesis, progression, and treatment response (39). By comparing scRNA-seq data from DLBCL patients and control samples, Xiaofei Ye et al. identified a CD70-CD27 interaction between malignant B cells and T cells. They also discovered that co-inhibitory signaling through TIM3 and TIGIT may be a major driver of T cell exhaustion and highlighted that HBV infection could influence DLBCL progression by promoting malignant cell survival or inducing immune escape (48). Together, these studies highlight the intricate interactions between immune and tumor cells within the DLBCL microenvironment, offering valuable insights for the design of therapies targeting the microenvironment.

### 3.3 Mechanisms of resistance

ScRNA-seq technology has made significant progress in uncovering the mechanisms of resistance in DLBCL. Xiyue Xu et al. employed scRNA-seq to comprehensively analyze the genomic features of the CD58 in DLBCL patients. Their study revealed that CD58 deletion could increase PD-L1 and IDO expression by activating the LYN/CD22/SHP1 signaling pathway, thereby facilitating immune evasion and contributing to treatment resistance (40). In terms of T-cell function regulation, Tingting Zhang et al. proposed that targeting the PD-1/PD-L1/L2 pathway in combination with CD73/A2aR could reverse T-cell dysfunction, providing a new therapeutic strategy for DLBCL (49). Additionally, Zi-Xun Yan et al. explored the potential mechanisms of resistance to CAR-T therapy in DLBCL patients through scRNA-seq. They discovered that cholesterol efflux in M2 macrophages might induce CD8+ T-cell exhaustion, thereby impairing CAR-T cell anti-tumor responses. The study suggested that cholesterol-lowering drugs or antibodies targeting HAVCR2 or TIGIT, when combined with CAR-T therapy, might enhance therapeutic efficacy (50).

Epigenetic regulation has also provided new insights for overcoming resistance in DLBCL. Oliver H. Krämer and Günter Schneider reviewed the potential of combining c-FOS and HDAC inhibitors (HDACi) in DLBCL, noting that this combined strategy might enhance anti-tumor efficacy through the modulation of epigenetic modifications (51). Further studies revealed that the potent HDACi LAQ824 could effectively kill DLBCL cells, and when used in combination with a c-Fos inhibitor, it significantly enhanced anti-tumor activity, offering a new direction for HDACi combination therapy (52).

In targeted therapy, Syahru Agung Setiawan et al. found that the BTK inhibitor zanubrutinib, in combination with the BCL-2 inhibitor navitoclax, synergistically suppressed double-hit lymphoma by inducing apoptosis and ferroptosis, providing a new combination therapy strategy to overcome DLBCL resistance (53). Additionally, the mechanism of bendamustine-rituximab (BR) therapy in DLBCL partially relied on apoptosis and enhanced immune responses induced by the cGAS-STING pathway, providing a conceptual framework for the clinical use of BR therapy (54).

In conclusion, these studies have identified key resistance mechanisms in DLBCL, including immune evasion, epigenetic alterations, and dysregulated signaling pathways, which provide critical insights for the development of combination therapies. By integrating scRNA-seq with functional assays such as CRISPR screens, a deeper understanding of these mechanisms is achieved, enabling the identification and validation of potential therapeutic targets (55). ScRNA-seq uncovers gene mutations and pathway changes, while CRISPR screens facilitate the functional validation of these targets, offering a promising strategy for therapeutic advancement.

### 3.4 Prognostic markers

ScRNA-seq technology has advanced significantly in the investigation of DLBCL prognostic indicators, revealing a close relationship between various cell types and molecular features with patient prognosis.

In terms of B lymphocytes, Zijun Y. Xu-Monette et al. found that the prognosis of DLBCL patients was substantially correlated with the abundance of tumor-infiltrating B lymphocytes (TIL-B), with patients with high TIL-B abundance showing larger proportions of memory B cells and naive CD4 T cells (56).

In T cells, Sylvia Zöphel et al. showed that CD16+ T cell populations had a protective role, with higher CD16+ T cell counts correlating with better prognosis, suggesting their potentialas markers for progression-free survival in aggressive B-NHL,including DLBCL (41). T-cell dysfunction was identified as a core mechanism of immune evasion and CAR-T therapy failure in DLBCL. Jinrong Zhao pointed out that intrinsic defects in CAR-T cells were one of the reasons for the variation in treatment efficacy (57). However, while these markers offer promising insights, their clinical validation in larger cohorts is still needed. Jin Jin et al., through scRNA-seq, discovered that elevated levels of Ki67, CD57, and TIM3, along with decreased CD69 levels in T cells, were associated with poor prognosis (58). Yao Wang et al. further found that a high proportion of CD8+ CAR-T cells and enhanced activation of CD8+ stem cell-like memory T cell populations were key to prolonging clinical efficacy, while the absence of CCR7 gene expression might explain the variability in CAR-T therapy outcomes (59). Qiqi Zhu et al., through scRNA-seq, revealed that the TIM3/Galectin-9 pathway induces exhaustion in CD8+ tumor-infiltrating lymphocytes, which is associated with immune suppression, poor prognosis, and a reduced response to CHOP chemotherapy (60). Nianping Liu et al., analyzing the tumor microenvironment of primary central nervous system DLBCL (PCNS DLBCL), found that a plasmablast-like program was linked to worse prognosis, with a higher score of exhausted CD8 T cells possibly contributing to poor outcomes (61).

In macrophages, their polarization status was closely associated with prognosis in DLBCL. Baoping Guo developed a prognostic model based on M2 macrophage-related genes (MS4A4A, CCL13, LTB, etc.), finding that patients in the high-risk group had poorer prognosis but were more sensitive to chemotherapy drugs and immune checkpoint inhibitors (62). Guangcan Gao et al. discovered that high expression of C1qC M2 macrophages predicted poor prognosis, with C1qC expression positively correlating with immune checkpoint molecules (63). Min Liu et al., through comprehensive transcriptomic analysis of macrophages in reactive lymphoid tissue (RLT) and different spatial regions of DLBCL, revealed transcriptomic differences in macrophages between the two, and established six macrophage signature profiles (MacroSigs) from distinct spatial sources. They found that specific MacroSigs were closely associated with the cellular subtypes of DLBCL and patient survival rates (64). These findings suggest potential therapeutic implications, but their clinical significance in guiding treatment strategies still requires robust validation.

In monocytes, Juliette Ferrant et al. identified S100A9^high monocytes as potential biomarkers for DLBCL, suggesting their role in tumor progression (65). Additionally, elevated expression of S100A8 was associated with poor prognosis in DLBCL, and inhibiting S100A8 was found to promote apoptosis and suppress tumor growth (66).

The role of other immune-related biomarkers in DLBCL prognosis has also attracted significant attention. Other immune-related biomarkers, such as CD161 monoclonal antibodies identified by Francesca Alvarez Calderon, have also shown potential for improving prognosis (67), but their clinical application is yet to be established. Martin A. Rivas et al. demonstrated that the cohesin complex played a key role in lymphoma development, and reduced expression of its subunits was linked to poorer prognosis in DLBCL patients (68). Jing Tang et al. developed a prognostic feature model based on exocytosis-related molecules, including SNRPB and CEP290, and confirmed the predictive capability of this model through immunohistochemistry (69). Additionally, Jurriaan Brouwer-Visser et al. found that CD20 expression loss in patients with relapsed/refractory B-cell non-Hodgkin lymphoma treated with Odronextamab could be a potential mechanism of resistance (70). In relapsed/refractory (R/R) DLBCL, scRNA-seq analysis of peripheral blood mononuclear cell samples identified 12 biomarkers (CD82, CD55, CD36, CD63, CD59, IKZF1, CD69, CD163, CD14, CD226, CD84, and CD31) that were notably upregulated. These markers correlate with patient prognosis and may offer potential new targets for therapy in R/R DLBCL (71). These biomarkers, while promising, require validation in larger, diverse patient populations to determine their clinical relevance in guiding treatment decisions.

The dual-protein expression lymphoma (DUEL) diagnostic method, based on the co-expression of BCL2 and MYC at the single-cell level, was validated through multiplex immunofluorescence and dual immunohistochemistry. The study demonstrated that DUEL was an independent adverse prognostic factor for DLBCL patients, providing a reliable basis for identifying high-risk patients and developing new therapeutic strategies (72). Liyuan Dai et al. combined single-cell and spatial transcriptomics technologies to reveal that the glycolytic metabolic activity in highly malignant DLBCL cells was significantly increased. High-glycolysis tissues exhibited abundant IFN_TAMs and reduced CD8+ T cells, and glycolysis gene expression was positively correlated with tumor malignancy. Immunohistochemical analysis validated the prognostic potential of glycolytic biomarkers such as STMN1, ENO1, PKM, and CDK1 (73). Additionally, radiomics parameters showed potential in prognostic prediction for DLBCL. One study has found that high total metabolic tumor volume (TMTV) and low tumor-infiltrating (TI) CD4+ cell levels are independently associated with poorer prognosis. Combining TMTV with TI cell analysis enhances the accuracy of prognostic prediction (74), but these findings need to be integrated with clinical data to validate their prognostic value.

## 4 Conclusion

ScRNA-seq technologies have demonstrated remarkable potential in DLBCL research, revealing key insights into tumor heterogeneity, microenvironment interactions, resistance mechanisms, and prognostic biomarkers. These studies have not only provided new perspectives for understanding the biological characteristics of DLBCL but also laid a solid foundation for developing precision therapeutic strategies. However, further validation of these findings in clinical settings is needed. Future research should explore how integrative approaches, such as spatial transcriptomics or single-cell multi-omics, can refine our understanding of DLBCL pathogenesis and help identify novel therapeutic targets. Overall, scRNA-seq could be instrumental in advancing early diagnosis, treatment monitoring, and personalized therapy, ultimately enhancing the field of precision medicine for DLBCL.
